# Public Understanding and Expectations of Digital Health Evidence Generation: Focus Group Study

**DOI:** 10.2196/56523

**Published:** 2025-01-20

**Authors:** Paulina Bondaronek, Jingfeng Li, Henry W W Potts

**Affiliations:** 1 Institute of Health Informatics, University College London, 222 Euston Road, London, NW1 2DA, United Kingdom, 44 07742966769; 2 School of Social Sciences, University of Westminster, London, United Kingdom

**Keywords:** mobile apps, digital health, public expectations, evidence of effectiveness, health risk perception, effectiveness, health risk, health app, public health, well-being, public trust, diagnostic tools, safety, mobile phone

## Abstract

**Background:**

The rapid proliferation of health apps has not been matched by a comparable growth in scientific evaluations of their effectiveness, particularly for apps available to the public. This gap has prompted ongoing debate about the types of evidence necessary to validate health apps, especially as the perceived risk level varies from wellness tools to diagnostic aids. The perspectives of the general public, who are direct stakeholders, are notably underrepresented in discussions on digital health evidence generation.

**Objective:**

This study aimed to explore public understanding and expectations regarding the evidence required to demonstrate health apps’ effectiveness, including at varying levels of health risk.

**Methods:**

A total of 4 focus group discussions were held with UK residents aged 18 years and older, recruited through targeted advertisements to ensure demographic diversity. Participants discussed their views on evidence requirements for 5 hypothetical health apps, ranging from low-risk wellness apps to high-risk diagnostic tools. Focus groups were moderated using a structured guide, and data were analyzed using reflexive thematic analysis to extract common themes.

**Results:**

A total of 5 key themes were established: personal needs, app functionality, social approval, expectations of testing, and authority. Participants relied on personal experiences and social endorsements when judging the effectiveness of low-risk digital health interventions, while making minimal reference to traditional scientific evidence. However, as the perceived risk of an app increased, there was a noticeable shift toward preferring evidence from authoritative sources, such as government or National Health Service endorsements.

**Conclusions:**

The public have a preference for evidence that resonates on a personal level, but also show a heightened demand for authoritative guidance as the potential risk of digital health interventions increases. These perspectives should guide developers, regulators, and policy makers as they balance how to achieve innovation, safety, and public trust in the digital health landscape. Engaging the public in evidence-generation processes and ensuring transparency in app functionality and testing can bridge the gap between public expectations and regulatory standards, fostering trust in digital health technologies.

## Introduction

The proliferation of health and wellness apps is substantial [1,2], with over 318,000 found in a 2018 study [3]. However, there is a wide gap between the availability and evaluation of digital health, with most publicly available apps not having been evaluated in the scientific literature [3]. They are typically rapidly developed and implemented with little formal evaluation to support their impact claims [4-6]. Many apps have proven not to be effective [7-10].

There are many reasons for this evaluation gap. The rapid advancement of digital health technologies has outpaced the development of comprehensive guidance. Governments have not wanted to hold back innovation around new technologies that promise to reduce health care costs and support economic growth. The dividing line between what should require effectiveness testing and what need not is unclear. This has all contributed to a gray area or “wild west” of regulation, a lack of clear guidelines, standards, and regulatory frameworks for evaluating digital health interventions. Stakeholders, including developers, health care providers, regulators, and users, face difficulties in navigating this uncertain terrain and ensuring that digital products and services meet appropriate standards of evidence.

What evaluation may be appropriate will vary depending on the digital technology under consideration. The National Institute for Health and Care Excellence Evidence Standards Framework (NICE ESF) [11], for example, offers recommendations of different study methods for different technology types, broadly based on a risk stratification [11]. A distinction is sometimes made between “digital therapeutics,” seen as akin to drug treatments and thus coming with rigorous evaluation [12], and “well-being apps,” perhaps closer to a self-help book or going to a yoga class and not requiring the same oversight. Well-being apps are seen as less of a concern for various reasons. The direct risks associated with them are small compared with a diagnostic or therapeutic tool. However, we note there can be a significant opportunity cost to public health apps that do not work. If someone uses, for example, a smoking cessation app that is not effective, they may be less inclined to try another that would have worked.

While there have been initiatives to improve the situation (including in the United Kingdom with the NICE ESF and Evaluating Digital Health Products [11,13]; and elsewhere, for example, DiGA (Digitale Gesundheitsanwendungen) in Germany [14]), there is a live debate over what evaluation is appropriate for what digital health products. However, we have seen little in this literature asking what the populace may want. Do the public expect or want more or less evaluation of digital health products?

Public policy [15,16] and research [17] both profess the value of public participation. The technical nature of clinical research, however, presents challenges. Public understanding of clinical research can be poor, although people’s perception of their own knowledge is higher [18]. Studies have focused on public understanding of randomized clinical trials, which is generally low, with ideas such as randomization and double blinding being challenging [19], as has been the case for many years [20]. This is significant as poor understanding undermines trial recruitment and public support for research, and may lead to people making poorer treatment choices [21,22].

Expectations of evidence generation will also relate to the public’s broader understanding of and belief in digital health. Policy makers may have techno-utopian views, seeing digital interventions as a solution to challenges in health care [23] through a revolution in the collection and use of data [24]. However, the public can be more cautious, being fairly positive about the potential for digital health, but with some concerns, particularly around safety, privacy, and depersonalization through the loss of human contact [25-28]. Attitudes can vary with the novelty and form of the technology involved [26].

Given all this, we aimed to explore the public’s understanding, expectations, and preferences regarding the evidence needed to demonstrate the effectiveness of digital health products and services. We also wanted to see how views vary by the nature of the digital health technology, particularly those that are seen as low-risk (well-being app) versus high-risk (diagnostic and therapeutic tools). We chose a focus group method as we thought the discursive nature of focus groups would help people consider a topic that may be relatively novel for them.

## Methods

### Sample

The research was carried out at the University of Westminster, and we sought to recruit a sample representative of the local population. We designed a recruitment poster outlining the content of the study and how to participate. The study was then advertised on social media (Twitter [subsequently rebranded X] and LinkedIn [Microsoft]) and through the University of Westminster’s Black and minority ethnic community engagement, the Black and Minority Ethnic All Members Network.

The inclusion criteria were UK residents aged 18 years and older. A sample framework was used to monitor the recruitment to ensure a diverse sample of participants. Interested participants were asked to fill out screening questions including age, gender, and ethnicity. We assessed levels of digital literacy with 2 previously used questions [29], asking “How difficult is it to use your smartphone [AND] install mobile applications/apps on your smartphone without someone else helping you?”, answered on a Likert scale. Participants could choose 1 of 4 meeting times for online focus groups. A total of 26 individuals contacted us, with 21 participants participating, divided into the 4 focus groups. Each participant received a participant information sheet and was told about the study goals and process and what they needed to do. If they had questions about the study, they were told they could ask them directly by email and get answers. The focus group interviews were conducted on July 11-July 12, 2023, through the University of Westminster’s Microsoft Teams account. Each focus group lasted 90 minutes.

### Ethical Considerations

The study was approved by the Psychology Ethics Committee at the University of Westminster (reference: ETH2223-3175). Participants received a Participant Information Sheet, provided written informed consent (Multimedia Appendix 1) through email, and could ask questions at any stage. Data were anonymized, securely stored, and handled in compliance with institutional data protection policies. Participants received vouchers valued at £20 (US $25.37) each to thank them for participating in the study. They were also provided with a summary of the interview results afterwards and given the opportunity to check and comment.

### Data Collection

The focus groups used a moderator’s guide. All groups were led by the same researcher (PB) for consistency. The other 2 authors attended all focus groups and took notes. The groups were recorded. We used the Microsoft Teams transcription as a starting point and then corrected this.

The goal throughout the focus groups was to explore participants’ perspectives on the evidence or proof required to demonstrate the effectiveness and accuracy of different types of digital health interventions. The moderator used a Microsoft PowerPoint presentation to guide the focus group process. We started the groups by introducing our definition of digital health, providing examples of 3 different technologies—medical imaging in hospitals, treating mental health at home, and improving fitness—to illustrate the broad spectrum of digital health. We then defined what we meant by evidence, which we loosely defined as “information or facts that support or prove something.” We then introduced 5 hypothetical digital health scenarios (alternating the order of scenarios for different groups: 1‐5; 5‐1).

The 5 hypothetical digital health scenarios and how we described them are given in Textbox 1.

These scenarios were selected and presented in a hierarchy (reversed for half the groups) to cover the spectrum of potential safety implications. We were informed in our choice by the NICE ESF, but included more well-being apps as these are not covered by the NICE ESF.

Textbox 1. **Five hypothetical digital health scenarios to guide the focus group.**

**Fitness app**

- Focuses on promoting good health, specifically for running.
- Tracks running activities—maps the run, records your speed, and logs running session

**Fitness app with artificial intelligence**

- Same as the fitness app
- Uses sensors to track your heart rate
- Uses artificial intelligence to then make a personalized recommendation as to when or how much you should run based on your age, weight, and heart rate

**Mindfulness app**

- Helps you deal with everyday stress
- Guided relaxation sessions
- Tracks how you are feeling

**Depression treatment app**

- Designed for people who have been diagnosed by a doctor as having clinical depression
- Offers a treatment
- Includes thinking exercises for the user to practice

**Skin cancer diagnosis app**

- Allows users to take a picture of their skin, specifically moles, for a potential skin cancer diagnosis
- Provides a diagnosis of skin cancer from the picture

### Data Analysis

We adopted an interpretivist epistemological framework and a social constructionism ontological stance. We used a reflexive thematic analysis [30,31] to interpret recurring themes based on the collected data and generate themes during coding. We used Reflexive Thematic Analysis Reporting Guidelines to guide the reporting of the analysis [32]. We anonymized the transcripts of the 4 focus groups, assigning a code to each participant. We also compared the transcriptions with our contemporary notes. We generated initial codes and applied them to the data, using Microsoft Excel sheets to identify common ground in participants’ expectations, concerns, and views on the effectiveness of digital health products. The codes were reviewed through repeated reading and analysis of the overall data. We also paid attention to how the codes related to the different scenarios presented. A total of 5 themes were eventually developed, which we present on a continuum. The codes and themes were reviewed again. Finally, we drew a thematic map for each scenario. We compared the commonalities and differences encoded between the thematic maps for each scenario, with respect to the hierarchy of scenarios (low-risk to high-risk digital health interventions) and our continuum of themes (from individual to community to authority).

## Results

### Participants

We used a semi–open-ended questionnaire to screen participants. Gender, age, and ethnicity were asked as open-ended questions to provide participants the flexibility of answer (Table 1). In total, 21 people (8 women and 13 men), aged 21‐50 years, participated in 4 focus groups. In the sample, 7 identified as Black British, 8 as Black other, 3 as White British, 2 as Asian, and 1 as Mixed. Everyone had access to smartphones, but 2 participants reported sometimes having difficulty installing software on their phones. Furthermore, 14 participants were in employment.

**Table 1.** Sample characteristics including gender, age, ethnicity, smartphone usage difficulty, and employment status (gender, age, and ethnicity were collected using open-ended questions).

Characteristic		Total (N=21), n
**Self-reported gender**		
	Women	8
	Men	13
**Age group (years)**		
	18‐25	5
	26‐35	13
	36‐45	2
	46‐55	1
**Ethnicity**		
	Black British	7
	Black other	8
	Asian	2
	White British	3
	Mixed	1
**Use smartphone**		
	Not difficult	21
**Install mobile apps**		
	Not difficult	19
	Somewhat difficult	2
**Employment status**		
	Full-time paid job (31+ hours)	9
	Part-time paid job (<31 hours)	5
	Doing paid work on a self-employed basis or within one’s own business	4
	Out of work (more than 6 months)	1
	Unpaid work for a business, community or voluntary organization	1
	Prefer not to say	1

### Overview

**Figure 1.**
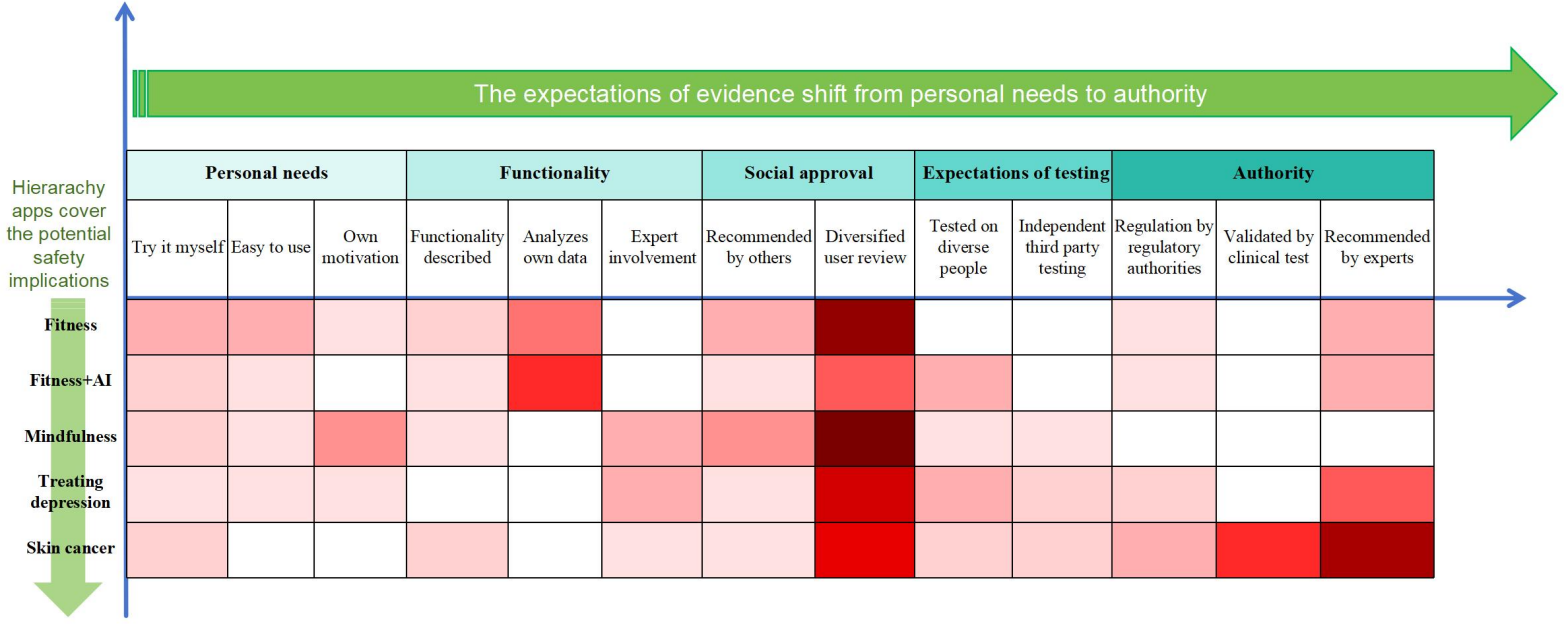
The expectations of evidence from different scenarios. The heatmap shows the approximate strength and frequency of participants’ comments with respect to each scenario, but should not be interpreted as a quantitative analysis. This heatmap shows public expectations for evidence across 5 digital health app types, progressing from low to high risk (fitness to skin cancer diagnosis). Themes—personal needs, functionality, social approval, testing expectations, and authority—are shaded to indicate how frequently each was mentioned. Darker shades represent higher frequency, with expectations shifting from personal experience for low-risk apps to clinical validation for high-risk apps. AI: artificial intelligence.

While we had sought to discuss evidence generation from a regulatory or research perspective, the participants often took a different perspective, discussing what factors influence their choice and use of digital health products and services. The 13 factors were eventually selected and grouped into 5 themes—personal needs, app functionality, social approval, testing expectations, and authority. Figure 1 shows the hierarchy of the 5 themes, from individual to community to authority. The color represents how often each factor was mentioned by the participants in the app scenario with different risk coefficients. The darker the color, the higher the frequency. White means not mentioned.

Across our hierarchy of 5 different digital health products, participants’ views changed. As potential health risks increased, there was an increased call for greater testing, and the involvement of authorities and health care professionals. For example, with the skin cancer diagnosis apps, participants preferred digital health products to be recommended by professionals and to be checked with hospital diagnoses.

### Personal Needs

Most participants first considered trying a digital health product themselves to see if it works for them. They generally reported believing they know their own physical and mental conditions best. Respondents explained that everyone is different, and what works for others may not work for them, so using an app yourself and checking effectiveness was reported to be one of the most reliable ways of evaluating it. Similarly, some participants thought they would only recommend the product to others after using it themselves successfully.

For example, with the fitness app, some participants said they would see the app as effective if they see positive changes in their body after using it:

*If I were using the fitness app, I would want probably milestones probably after a week after two weeks to three, I should get changes and stuff like that.*

[P18]

With the fitness app with artificial intelligence (AI), participants discussed whether the AI’s recommendations could be trusted. As P11 said,

*If I’ve used it a few times and it’s working for me. Then you begin to build up trust, or this is what it can do, and this is what it has trouble doing*.

[P11]

With the mindfulness app, as this is related to their daily mental state, participants felt more need to try it for themselves:

*Like people every day, stress is different like stress is different for everybody, like what might be stressful for me might not be stressful for another individual.*

[P18]

Similarly, with the depression treatment app:

*I think I’ll just go on and try it out on my own.*

[P21]

Factors, such as ease of use and personal motivation, were highlighted, with many participants expressing that these aspects greatly influence their willingness to adopt and continue using digital health apps. Ease of use seemed to be a fundamental prerequisite for participants.

When talking about personal motivation to use an app, P3 explained,

*There are many fitness apps. You gotta find the one you really like.*

[P3]

When participants felt an app was what they wanted, they would be motivated to continue using it. For mindfulness and depression apps, some participants said that if the app made them feel better, that would mean the app was effective. They would then increase their motivation and become more committed to using the app:

*I know it’s different for everyone and depending, like the other person said, depending on how invested you are in using the app, that’s how much of the result you see.*

[P14]

### Functionality

Participants saw a product’s functionality as a determining factor of whether it is effective or not. Before using the fitness or fitness apps with AI, some participants said they would check online information about the product to understand its functions and effects. With the higher risk products, such as the skin cancer diagnosis app, some participants felt that developers demonstrating to the public how the app works could be evidence of the app’s effectiveness, such as having videos or explaining within the app how it works. A product’s diversity in functions was also seen to be related to its ability to work for diverse populations. For fitness apps, some participants talked about different levels of exercise being supported to meet the needs of different users, and how apps should also have the ability to collect physiological data and analyze data:

*The application needs to identify the users you know to get data and then be able to process the data and give you a well-informed recommendation.*

[P12]

For the other apps, participants wanted to have experts involved in the apps, and for the apps’ features to allow users to connect with experts, which would increase their trust in using the app. For example, they wanted the depression treatment app to entail further follow-up by experts according to the degree of depression. Talking about the skin cancer diagnosis app, P19 explained:

*It will be my only safety because it depends on the professionals attached, whatever the app wants to support.*

[P19]

### Social Approval

Most participants mentioned social approval as a strong influence on their belief in the effectiveness of an app. They described recommendations from friends and relatives as being critical when using an app. This is, in part, because they have a high degree of familiarity with relatives and friends, know their lifestyles and physical conditions, and this therefore allows them to put relatives’ and friends’ recommendations in context. As a result, across all the apps, participants trusted the recommendations of relatives and friends more:

*For instance, if I’m into a particular type of job and the person is also into that type. I could read what everyone’s stress goes through together with that, and I could greatly do the same. I could use that as evidence, and I trust such an app*

[P18]

After recommendations from friends and family, user reviews were the main factor influencing participants to choose an app. For apps with varying health risks, the user reviews that people wanted to see differed. Participants said they would look at overall user reviews before downloading the fitness app to check its effectiveness. This would make it easier for them to understand what the app does and whether it is right for them. Participants believed that the more people who downloaded and positively reviewed an app, the more likely it was to be effective. Therefore, participants also mentioned the number of downloads as a source of evidence. While discussing the fitness app with AI, 1 participant questioned the authenticity of reviews. Some participants tended to ask questions in the app user’s comments section and then use the subsequent interaction and answers from those users to help them decide whether to choose an app:

*I need evidence cos it’s already acting in place of my doctor and already giving me recommendations… I tend to stop and pause for reactions from people and get reviews.*

[P17]

Discussing the mindfulness app, some participants felt they needed feedback from people close to them, such as observing how their friends used it to see how the app worked. As for the app to treat depression, 1 participant mentioned that the stories people share on social media are also a way for users to comment. Since they are real user experiences, this kind of comment has impact because

*we buy because of what people have said, how it’s done.*

[P11]

Participants found the comments of those who suffer from depression more persuasive, as P9 explained:

*Seeing that evidence from the person who used this app, the person was suffering from depression, and this kind of treatment covered. So I’ll need such things to use this kind of app.*

[P9]

Participants wanted to know if different people had used the app and it had worked for them. Different people could refer to differences in ethnicity, sex, age, and lifestyle. Some participants wanted to see physical evidence of results from fitness apps, such as outcomes like weight loss and faster running. We pushed participants to specify the number of people from whom they would like to have some feedback. Many participants considered the experience of 1‐5 people enough to demonstrate effectiveness.

For the AI function of the fitness app, participants were concerned about whether the AI could make accurate recommendations based on the age and gender of different people. Mindfulness apps, they said, need to have been used and tested for effectiveness by various different people, and the number of participants who brought this up was larger than with the other apps. Some participants believed that different lifestyles lead to varying stress levels:

*I think they have different needs, especially for mindfulness apps. The option to choose a lifestyle would be helpful.*

[P3]

Here, participants talked of how 5 or more people’s reviews could be used as evidence, with feedback wanted from people with differing levels of depression:

*If the treatment can cater for different people depending on their diagnosis… other people have used them for treatments, and it’s a success for them. I think I’d be OK to use it.*

[P14]

### Expectations of Testing

Some participants mentioned that developers should conduct tests on different people before launching digital health products and services. What participants meant by different people again refered to differences in various aspects, such as different ethnicities, ages, genders, lifestyles, and health conditions. Depending on the app, participants felt that the focus of the test should vary. P1 believed that the fitness app with AI should also implement

*Background checks and testing. For a different age group and probably put in some information.*

[P1]

P20 thought mindfulness apps *“should cater to various individuals.”* The participants did not evince any familiarity with the details of scientific evaluations.

Being diagnosed with cancer was recognized as having a significant impact on people’s lives. Thus, some participants mentioned that the testing for a skin cancer diagnosis app should be mainly to determine the app’s accuracy and to improve the richness of the test data. In terms of testing being on a diverse group, for skin cancer diagnosis apps, participants were concerned about differences in skin color and wanted to see more usage data than for other apps. Furthermore, 1 participant suggested testing on 20 people, 5 of whom had the same skin color as himself.

For apps to treat depression, it was suggested that testing should be conducted strictly following academic experiments to test actual effectiveness:

*I’m expecting that this app has been used for more, more like a randomised set of people before it was rolled out, and then, you know, a study should be carried out on them before and after the intervention. Then you compare how well, how effective they there’s been. So, for me, I think that would be the evidence that we’re looking for.*

[P15]

Some participants said that the testing of digital health products should be carried out by third-party institutions. This should, first, be an authority in a relevant professional field, such as the National Health Service (NHS) or other professional bodies. Second, it should be independent and unrelated to the developer’s business operations. In addition, 1 participant noted for depression apps that such third-party tests will be more trustworthy than the developers’ own explanations. Another participant said that skin cancer diagnosis app testing should be used in a real-life setting:

*I also think the app should have been used to diagnose real-life diseases in some trials before the agency certified it for launch.*

[P15]

Information on testing should be made available, for example, within the app. Discussing the skin cancer diagnosis app, a participant said,

*I think the app should contain information about the testing and trials done to ascertain this app’s effectiveness.*

[P15]

### Authority

Participants mentioned the need for government and NHS regulation for all the apps. Participants believed that government and NHS regulation would increase their trust in the apps’ effectiveness. A digital health product vetted by the government or NHS was taken to mean that it has been tried by many people and has worked. Government regulation was seen as most important for skin cancer diagnosis apps because of the *“severity of the condition”* [P20] and the need to avoid misdiagnosis.

Participants felt it was necessary that the skin cancer app’s diagnoses were compared with hospital diagnoses to determine the app’s accuracy. It was the only app where participants talked about a comparison with a hospital diagnosis during the discussions. To avoid misdiagnosis, participants would not unilaterally believe the app diagnosis:

*I also think any form of diagnosis that should be done with this app should also be related to the GP and others for clarification to get a better result.*

[P13]

The use of the app, they said, should only be an intermediate process, and the effectiveness of the app would ultimately be proved by the hospital diagnosis.

Participants rated professional recommendations as being necessary for the other 4 apps. For fitness apps, recommending professionals referred to experts in this field, such as fitness coaches and sports athletes. For the skin cancer diagnosis app, most participants said only a doctor’s approval would lead them to use it. One participant stated,

*I would also be looking for certification from a cancer body.*

[P15]

For depression treatment apps, people would consult their doctors for recommendations:

*I would actually contact my doctor first and let him know about the app and if he was about to use it and can give me go ahead to use it.*

[P1]

Most participants believed that the government or NHS should regulate all digital health products:

*These apps must be censored and monitored because they involve our well-being. This application acts as a substitute for a visit to our GP. So, we need to be sure of how we administer the application and its reliability. About all the apps.*

[P17]

## Discussion

### Principal Results

This study aimed to explore participants’ trust in digital health technology and what evidence the public wants from digital health interventions for increasing health and well-being. Following the NICE ESF’s risk hierarchy, different scenarios were designed, including wellness apps and treatment and diagnostic apps. We found that (1) the public’s expectations for evidence of effectiveness are intertwined with the factors that influence their choice and use of digital health interventions; (2) the public’s expectations for evidence of effectiveness are based more on individual experiences and social approval than scientific evidence; (3) as the perceived risks associated with different apps increase, so to do the public’s expectations for the evidence of their effectiveness; and (4) there is a desire for recommendations or certifications from relevant bodies, particularly as the potential risk increases.

### Comparison With Previous Work

This study supports the findings that the public understanding of what constitutes high-quality research evidence is low [18-20]. As shown previously [21,22], this can lead to challenges in research support and affect the decision-making related to the digital health selection, uptake and engagement, behavior and, ultimately, health outcomes.

We also saw people exhibiting cautious optimism, recognizing potential benefits while maintaining a healthy scepticism about new technologies, as seen in previous studies [25,26].

This study found that the public prefers to verify the effectiveness of digital interventions through personal experience. There are parallels with some research on earlier generations of digital health technology. Herian and colleagues [33] found that greater experience with health care was associated with lower trust in government to regulate electronic health records, which suggests that personal experience was valued more. Several studies showed that people seeking health information online relied more on their own experience of websites than credibility assessments [34]. We know more generally that personal choice and personal recommendations are important in people’s engagement with digital health interventions [35].

This focus on personal experience supports the view of Pagoto and Bennett [36] that interface design is crucial in the development process of the app. The “person-based” approach emphasizes using qualitative research to understand the psychosocial background of a wide range of target populations during the development process of digital interventions and getting feedback from them to improve applicability and effectiveness [37]. This is consistent with the findings of this study that the public expects greater transparency in the development and testing process, which is beneficial for improving the social acceptance of digital interventions by testing with different people and validating the results.

### Implications

The study found that the public’s concept of evidence diverges significantly from the scientific community’s understanding. While researchers may refer to evidence as data collected from rigorous, controlled settings, such as randomized controlled trials, participants in our focus groups considered personal experiences and anecdotal successes as credible evidence for assessing digital health products.

Public policy [15,16] and research [17] both strongly endorse involving the public. This study calls for a systematic approach to involving the public in digital health evidence generation. To effectively engage the public, it is essential to grasp their level of understanding about the need for evidence generation in digital health and why it matters, that is, the potential for safety issues but also missed opportunities for prevention (for so-called lower risk digital health, such as apps for physical activity). This requires educational methods with a substantial instructive component as well as coproduction, such as citizen juries, and necessitates clear, accessible explanations of evidence generation, effectiveness, and the potential detrimental effects of digital health interventions that are widely used but not evaluated. Educating the public about the need for evidence generation in digital health could increase the pressure and motivation of developers to embed evidence generation practices in digital health. Equally, the pressure of the public may encourage regulators to require minimal evidence generation before publicly available digital health is introduced on the app distribution platforms.

Professional support and education can foster user engagement with digital health technology [38]. Social media platforms present opportunities for public communication and behavioral interventions [39]. Developers and relevant authorities can use diverse strategies, including leveraging social media, to enhance the efficacy of digital health apps for promoting public health behaviors. A detailed description of an app’s functionalities, effects, and target population should be provided so that individuals can assess its effectiveness quickly. Using documentaries and press releases can promote transparency in digital health product development and testing processes, enhancing public trust. Using social media platforms to share user reviews, such as inviting long-term clients from diverse backgrounds and age groups to tell their stories or engaging professionals like renowned doctors, experts, and athletes to provide insights, fosters societal acceptance. It is crucial to increase public understanding of the impact of the lack of evidence generation in digital health, such as safety concerns and missed opportunities for health promotion when popularity rather than evidence for effectiveness is the primary factor driving downloads.

### Strengths

To the best of our knowledge, this is the first study to explore public expectations and preferences regarding evidence generation for the effectiveness of digital health interventions—a stakeholder group that has been overlooked. The sampling strategy aimed to include those at risk of digital inequalities and included a range of ages, genders, socioeconomic statuses, and ethnicities, showcasing a broad spectrum of views. The various scenarios developed for this study were comprehensive.

### Limitations

While the study was open to all UK residents, we recruited mostly from London. With 21 participants, the study may not capture the full range of public opinions. The materials used to guide the focus groups and the results could be used to facilitate the development of a survey to get larger samples, which could provide more robust data. We offered a £20 (US $25.37) voucher, which might have influenced motivations to participate in this study.

### Conclusion

This study has explored public expectations regarding the evidence needed to demonstrate the effectiveness of digital health products and services. The public tends to favor evidence that is relatable and directly experienced, such as personal success stories, user reviews, and recommendations from significant others. The public made minimal reference to what researchers would perceive as rigorous evaluation methods. However, there was also support for authoritative recommendations and the independent testing of products. As health risks associated with digital health increased, there was a growing demand for more substantial evidence and authoritative recommendations from health care professionals.

## Supplementary material

10.2196/56523 Multimedia Appendix 1 Informed consent.

## Abbreviations

AI: artificial intelligence
DiGA: Digitale Gesundheitsanwendungen
NHS: National Health Service
NICE ESF: National Institute for Health and Care Excellence Evidence Standards Framework
