# Supplementary material for: Public Understanding and Expectations of Digital Health Evidence Generation: Focus Group Study
Source: JMIR Form Res. 2025 Jan 20;9:e56523. doi: 10.2196/56523 (PMC11769687; doi:10.2196/56523)
Supplement: Multimedia Appendix 1 [file formative-v9-e56523-s001.docx]

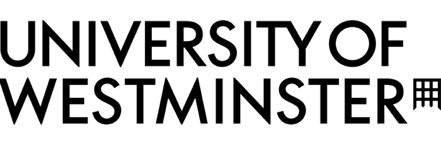
Consent form Version 1

**If you have any questions about the consent process, please feel free to contact the research team prior to completing it. In completing this consent form I am indicating that my participation has been explained to my satisfaction and I am agreeing to and understand the following.**

**Please check each box below to indicate your agreement:**

| My participation in this research is on an entirely voluntary basis. | □ |
| --- | --- |
| I am able to stop at any point during the process without having to provide an explanation. | □ |
| Once I have taken part, I am still able to withdraw my data at any point until the data has been anonymised or analysed as part of the research project. I understand that if I do not provide any identifying information it will not be possible to remove my data after I submit it as it will not be possible to identify it. | □ |
| I do not have to answer all questions or complete all tasks that are presented to me. | □ |
| My data will be anonymised and all identifying features will be removed so that my contribution will not be identifiable when reporting this research. | □ |
| If I provide any personal identity data this will be treated confidentially and in accordance with the University of Westminster ethical guidelines and British Psychological Society code of human research ethics. It will be securely stored and managed in accordance with the General Data Protection Regulation 2018 and the Data Protection Act 2018. | □ |
| The responses that I provide **may** be shared with members of the research and/or teaching team, and the University of Westminster External Examiner. | □ |
| The duty of confidentiality is **not absolute** and in exceptional circumstances this may be overridden by more compelling duties such as to protect individuals from harm. | □ |
| My anonymised contribution to this research may be used for future research and may undergo secondary analysis. Future research may be unrelated to the goals of this study and may be conducted by researchers that are unrelated to this study. | □ |
| I confirm that I am aged 18 years or older. | □ |
| I consent to take part in this research study. |  |
